# Supplementary material for: Post-translational amino acid conversion in photosystem II as a possible origin of photosynthetic oxygen evolution
Source: Nat Commun. 2022 Jul 21;13:4211. doi: 10.1038/s41467-022-31931-y (PMC9304363; doi:10.1038/s41467-022-31931-y)
Supplement: Supplementary file 1 — Supplementary Information [file 41467_2022_31931_MOESM1_ESM.pdf]

Post-translational amino acid conversion in photosystem II as a possible origin of photosynthetic oxygen evolution

Shimada *et al*.

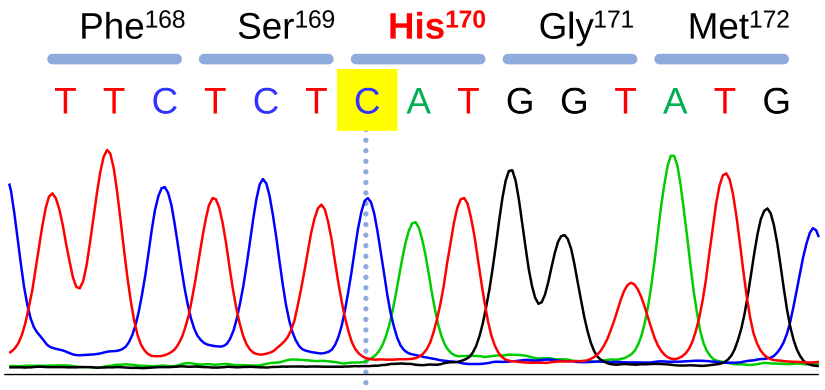

Supplementary Fig. 1. DNA sequence chromatogram of the cDNA of *psbA2* mRNA obtained from D1-D170H cells. Raw traces for G, C, A, and T are shown in black, blue, green, and red, respectively. Note that there is no band of G of the GAT/GAC codons (Asp) at the C position of the CAT codon of His170. The sequence of the cDNA was deposited in the DNA Data Bank of Japan (accession number: LC717798).

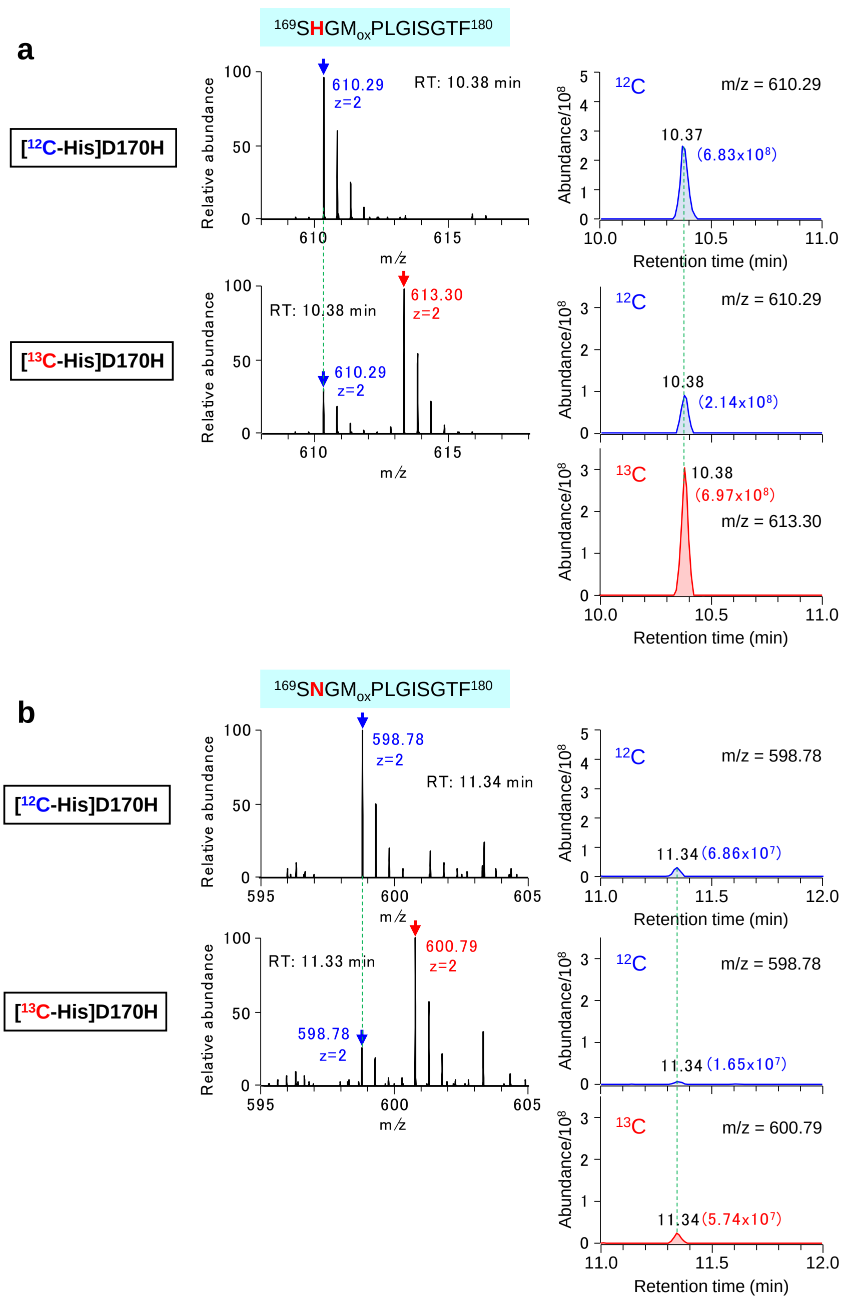

Supplementary Fig. 2. LC-MS analysis of the D1-D170H mutant. a, b, MS spectra (left) and MS chromatograms (right) of the polypeptide fragments of the D1 proteins (between S169 and F180 with oxidized M172), which have (**a**) His and (**b**) Asn at position 170, in the PSII core complexes isolated from [^{12}C-His]D170H and [^{13}C-His]D170H cells. A peak of the target polypeptide fragment in the MS spectrum is indicated by blue (^{12}C-H170/N170) or red (^{13}C-H170/N170) arrows. The area intensity of the chromatogram peak is given in parentheses. Source data are provided as a Source Data file.

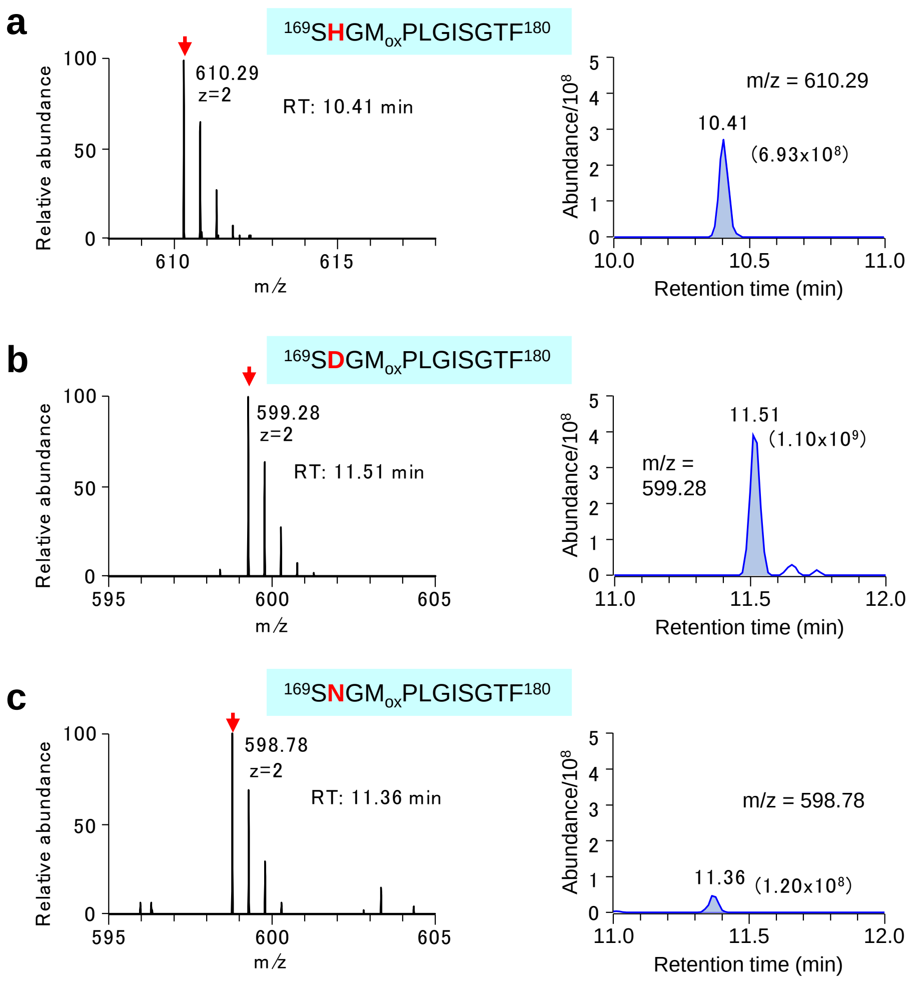

Supplementary Fig. 3. LC-MS analysis of the D1-D170H mutant grown under dim light. a, b, c, MS spectra (left) and MS chromatograms (right) of the polypeptide fragments of the D1 proteins (between S169 and F180 with oxidized M172), which have (**a**) His, (**b**) Asp, and (**c**) Asn at position 170, in the PSII core complexes isolated from D170H cells mixotrophically grown under dim light. A peak of the target polypeptide fragment in the MS spectrum is indicated by a red arrow. The area intensity of the chromatogram peak is given in parentheses. Source data are provided as a Source Data file.

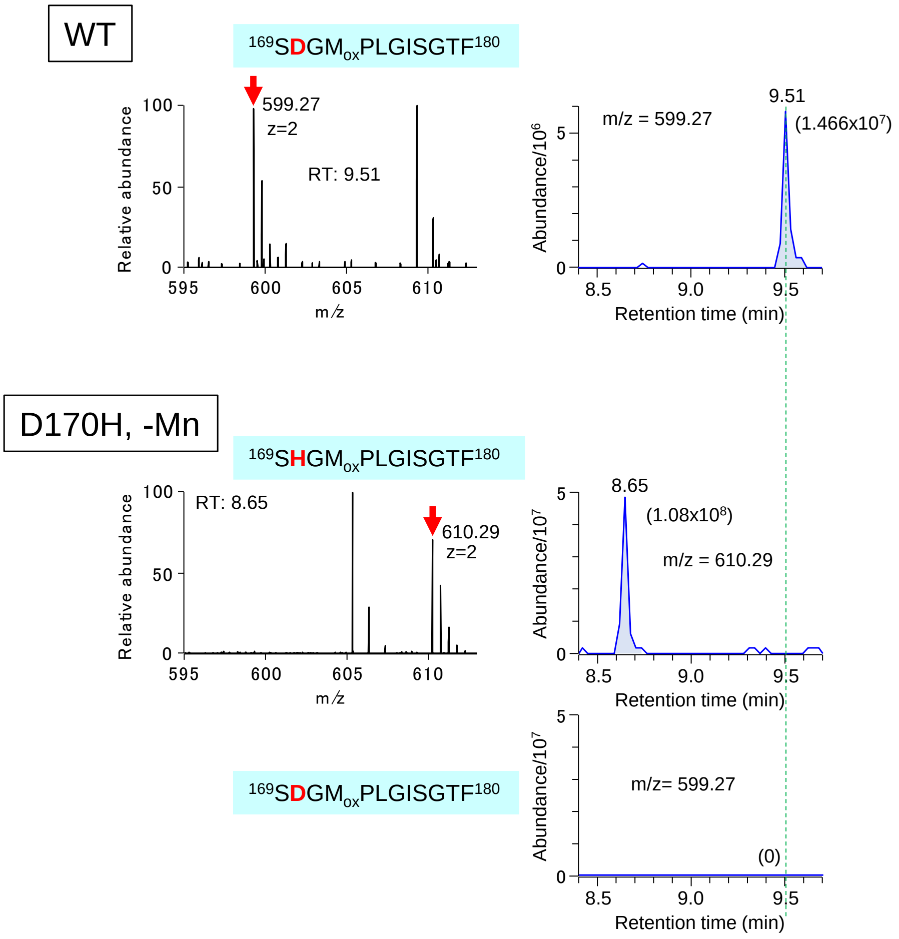

Supplementary Fig. 4. LC-MS analysis of the D1-D170H mutant grown in Mn^{2+}-depleted medium. MS spectra (left) and MS chromatograms (right) of the polypeptide fragments of the D1 proteins (between S169 and F180 with oxidized M172) in the PSII core complexes isolated from D170H cells mixotrophically grown in medium in the absence of Mn^{2+} (lower panel) in comparison with the data of WT* grown in normal medium (upper panel). A peak of the target polypeptide fragment in the MS spectrum is indicated by a red arrow. The area intensity of the chromatogram peak is given in parentheses. Source data are provided as a Source Data file.

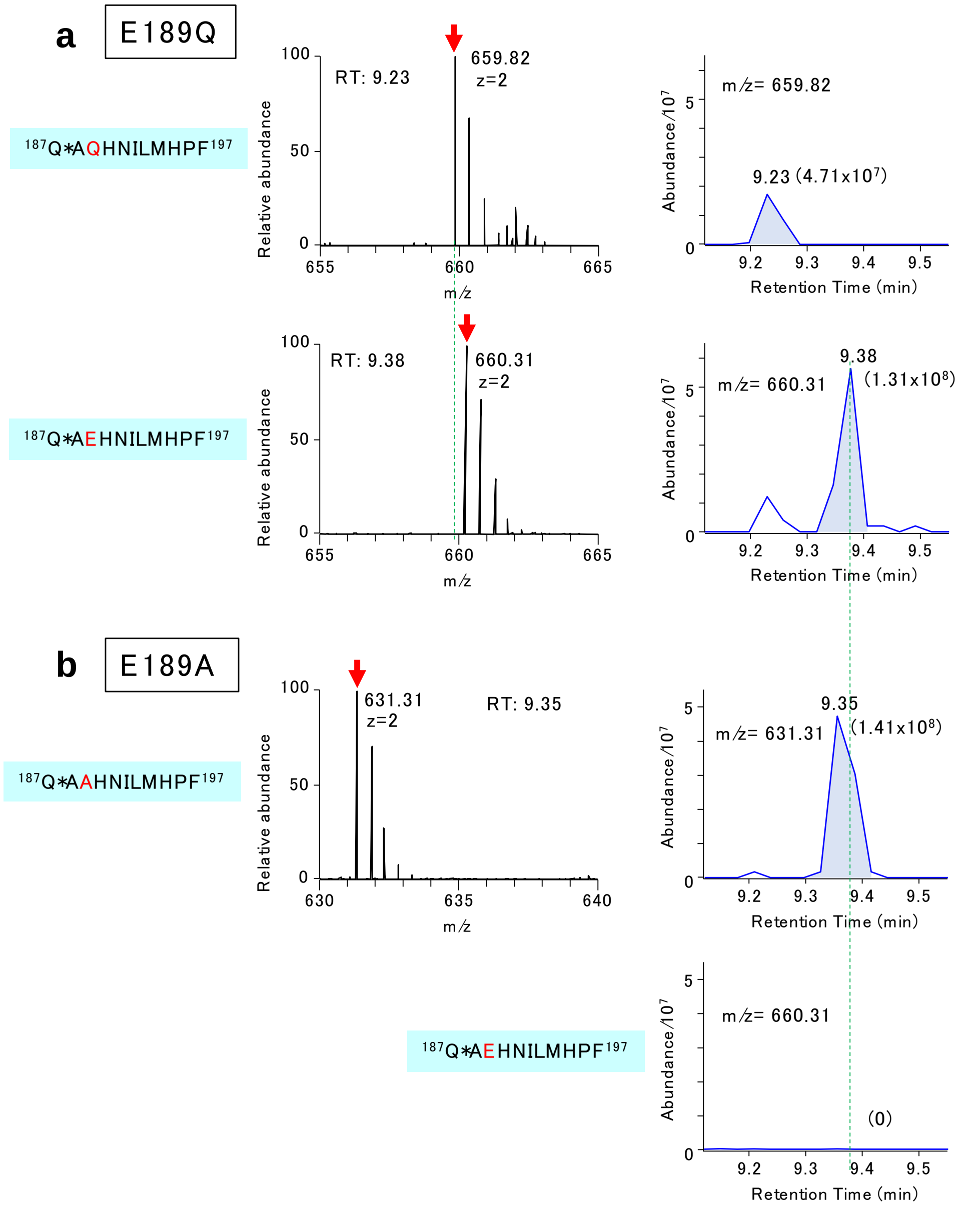

Supplementary Fig. 5. LC-MS analysis of the E189Q/A mutants. a, b, MS spectra (left) and MS chromatograms (right) of the polypeptide fragments of the D1 proteins (between Q187 and F197) in the PSII core complexes isolated from (**a**) E189Q and (**b**) E189A cells. Q187 in the fragment (marked with an asterisk) was modified to pyroglutamic acid. A peak of the target polypeptide fragment in the MS spectrum is indicated by a red arrow. The area intensity of the chromatogram peak is given in parentheses. Source data are provided as a Source Data file.

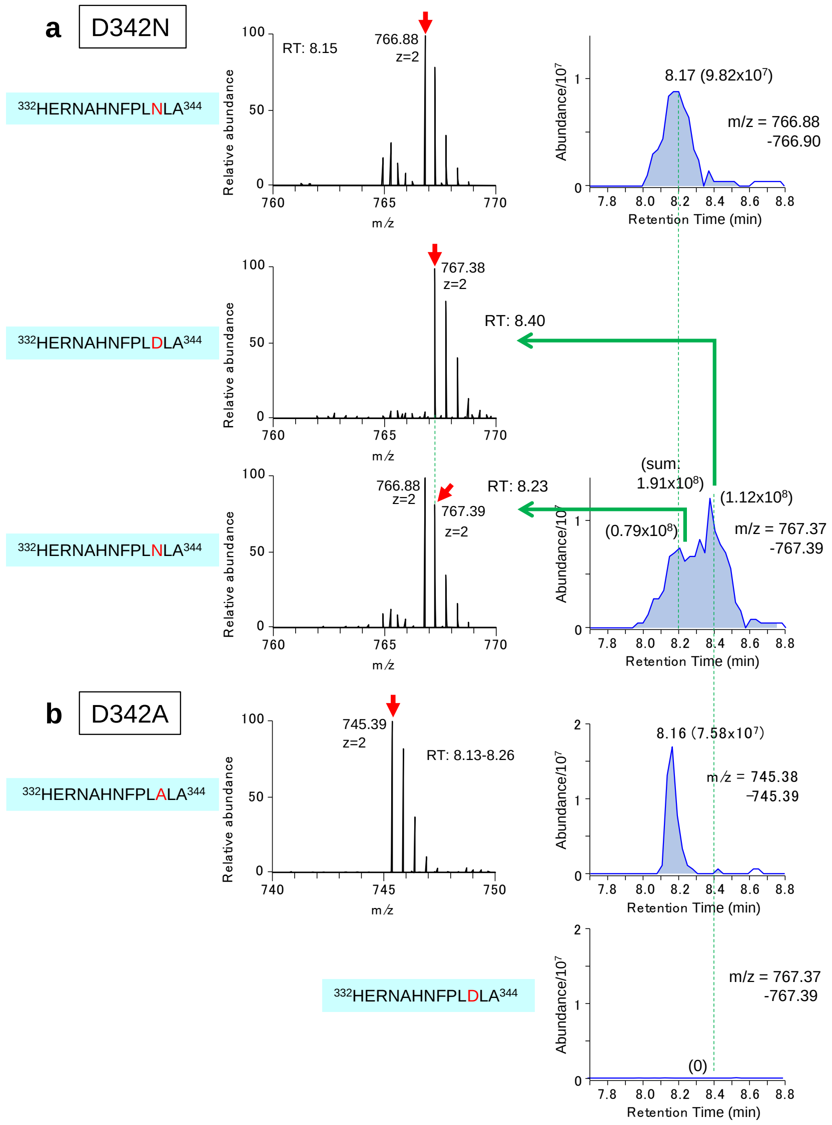

Supplementary Fig. 6. LC-MS analysis of the D342N/A mutants. a, b, MS spectra (left) and MS chromatograms (right) of the polypeptide fragments of the D1 proteins (between H332 and A344) in the PSII core complexes isolated from (**a**) D342N and (**b**) D342A cells. A peak of the target polypeptide fragment in the MS spectrum is indicated by a red arrow. The area intensity of the chromatogram peak is given in parentheses. In the D342N mutant, the MS peak of a fragment with D342 (m/z = 767.38) almost coincides with the isotope peak of a fragment with N342 (m/z = 767.39), and hence the two chromatogram bands corresponding to these fragments were observed. The area intensity of the fragment only with D342 (1.12×10^8) was estimated by subtraction of that of the fragment only with N342 (0.79×10^8), which was estimated from the intensity of the chromatogram band for the main MS peak at m/z = 766.88 (0.982×10^8; upper panel) multiplied by 0.80 (a relative intensity of the isotope peak), from the whole intensity of both the fragments (1.91×10^8). Source data are provided as a Source Data file.

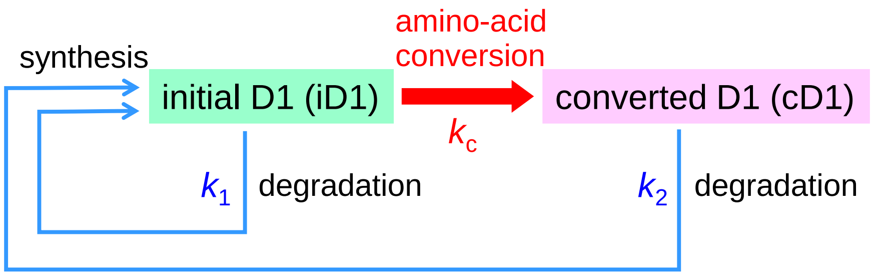

In steady state ([iD1], [cD1]: constant)
rate of synthesis of iD1
= rate of degradation of iD1 + rate of degradation of cD1
= k_1[iD1] + k_2[cD1]

$$\frac{d[\mathrm{iD1}]}{dt} = -(k_\mathrm{c} + k_1)[\mathrm{iD1}] + k_1[\mathrm{iD1}] + k_2[\mathrm{cD1}]$$
$$= -k_\mathrm{c}[\mathrm{iD1}] + k_2[\mathrm{cD1}] = 0$$
$$\frac{d[\mathrm{cD1}]}{dt} = k_\mathrm{c}[\mathrm{iD1}] - k_2[\mathrm{cD1}] = 0$$

Thus,

$$\frac{[\mathrm{cD1}]}{[\mathrm{iD1}]} = \frac{k_\mathrm{c}}{k_2}$$

Supplementary Fig. 7. Accumulation of the amino-acid converted D1 protein in the D1 turnover. Under the steady state, the amount of the converted D1 protein (cD1) relative to that of the initial D1 protein (iD1) is determined by the ratio of the rate constant of amino-acid conversion (k_c) and that of the degradation of cD1 (k_2). Note that the relative amounts of iD1 and cD1 are irrelevant to the rate constant of the degradation of iD1 (k_1).

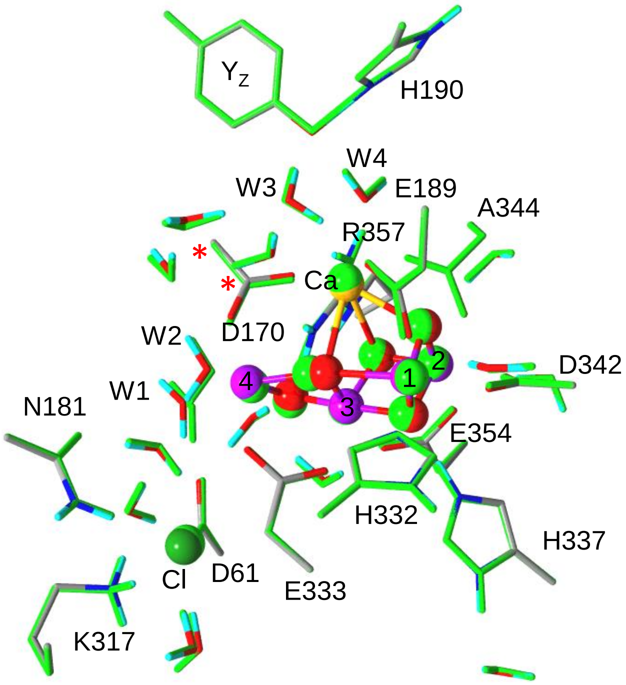

Supplementary Fig. 8. QM region in the QM/MM calculations. Optimized structures of the QM region in the QM/MM calculations of the OEC in the S_1 state (atomic color) and the S_2 state with oxidized Mn4 (green color). The carbon atoms labeled with ^{13}C in D1-D170 are marked with red asterisks. Amino acid residues are on the D1 protein except for K317 on the D2 protein and E354 and R357 on the CP43 protein. The numbering of the Mn atoms in the Mn cluster is also shown. Source data are provided as a Source Data file.
