# Supplementary material for: Post-translational amino acid conversion in photosystem II as a possible origin of photosynthetic oxygen evolution
Source: Nat Commun. 2022 Jul 21;13:4211. doi: 10.1038/s41467-022-31931-y (PMC9304363; doi:10.1038/s41467-022-31931-y)
Supplement: Supplementary file 3 — Reporting Summary [file 41467_2022_31931_MOESM3_ESM.pdf]

nature portfolio

Corresponding author(s): Takumi Noguchi

Last updated by author(s): Jul 7, 2022

Reporting Summary

Nature Portfolio wishes to improve the reproducibility of the work that we publish. This form provides structure for consistency and transparency in reporting. For further information on Nature Portfolio policies, see our Editorial Policies and the Editorial Policy Checklist.

Statistics

For all statistical analyses, confirm that the following items are present in the figure legend, table legend, main text, or Methods section.

n/a	Confirmed	
☐	☒	The exact sample size (n) for each experimental group/condition, given as a discrete number and unit of measurement
☐	☒	A statement on whether measurements were taken from distinct samples or whether the same sample was measured repeatedly
☒	☐	The statistical test(s) used AND whether they are one- or two-sided<br>*Only common tests should be described solely by name; describe more complex techniques in the Methods section.*
☒	☐	A description of all covariates tested
☒	☐	A description of any assumptions or corrections, such as tests of normality and adjustment for multiple comparisons
☐	☒	A full description of the statistical parameters including central tendency (e.g. means) or other basic estimates (e.g. regression coefficient) AND variation (e.g. standard deviation) or associated estimates of uncertainty (e.g. confidence intervals)
☒	☐	For null hypothesis testing, the test statistic (e.g. F, t, r) with confidence intervals, effect sizes, degrees of freedom and P value noted<br>*Give P values as exact values whenever suitable.*
☒	☐	For Bayesian analysis, information on the choice of priors and Markov chain Monte Carlo settings
☒	☐	For hierarchical and complex designs, identification of the appropriate level for tests and full reporting of outcomes
☒	☐	Estimates of effect sizes (e.g. Cohen's d, Pearson's r), indicating how they were calculated

Our web collection on statistics for biologists contains articles on many of the points above.

Software and code

Policy information about availability of computer code

Data collection	FTIR: OPUS 7.8
Data analysis	MS/MS spectra search: Matrix Science, MASCOT 2.7<br>Drawing of MS spectra and MS chromatograms: Thermo Fisher Scientific, Inc., FreeStyle 1.3 SP2 and Xcalibur 4.1.50, Qual Browser<br>QM/MM calculations: Gaussian 16 program package<br>Molecular mechanics calculations: Amber 16

For manuscripts utilizing custom algorithms or software that are central to the research but not yet described in published literature, software must be made available to editors and reviewers. We strongly encourage code deposition in a community repository (e.g. GitHub). See the Nature Portfolio guidelines for submitting code & software for further information.

Data

Policy information about availability of data

All manuscripts must include a data availability statement. This statement should provide the following information, where applicable:

- Accession codes, unique identifiers, or web links for publicly available datasets
- A description of any restrictions on data availability
- For clinical datasets or third party data, please ensure that the statement adheres to our policy

The data generated in this study are provided in the Source Data file. The sequences of cDNA of the psbA2 mRNA was deposited in the DNA Data Bank of Japan under accession number LC717798 (https://getentry.ddbj.nig.ac.jp/getentry/na/LC717798/). The coordinates of the PSII complex used in QM/MM calculations were obtained from PDB 4UB6 (http://doi.org/10.2210/pdb4UB6/pdb).

Field-specific reporting

Please select the one below that is the best fit for your research. If you are not sure, read the appropriate sections before making your selection.

- [x] Life sciences
- [ ] Behavioural & social sciences
- [ ] Ecological, evolutionary & environmental sciences

For a reference copy of the document with all sections, see nature.com/documents/nr-reporting-summary-flat.pdf

Life sciences study design

All studies must disclose on these points even when the disclosure is negative.

Sample size	No statistical methods were used to predetermine sample size. Sample sizes were determined based on the previous experiences and established standards. In measurement of O2 evolution activity, measurements of three distinctive samples are enough taking into account the standard deviation relative to the activity values (less than 0.1) of the D1-E189Q and D1-D342N PSII. In FTIR measurements, 100-160 spectra were coadded to provide enough signal-to-noise ratios (Debus et al., Biochemistry 2005; Kitajima-Ihara et al., Biochim. Biophys. Acta 2020). Identification of mass signals in LS-MS is not relevant to a sample size.
Data exclusions	No data were excluded.
Replication	In FTIR measurements, spectra of each mutant were measured a couple of times under the same or slightly different conditions (e.g., with and without bicarbonate), showing basically the same features. O2 evolving activities of the mutants were estimated in isolated PSII complexes in this study (n=3) and also in cells (n=3), providing the similar tendencies. In LC-MS analysis, the D1 protein of the D1-D170H mutant was analyzed several times for samples with the same or different growth conditions (photoautotroph, mixotroph, 12C-His incorporated etc.), while the D1 proteins from other mutants were examined in different buffers containing trifluoroacetic acid or formic acid (this study), all showing consistent results.
Randomization	Randomization is not relevant to this study, because we characterized the properties (FTIR spectra, O2 evolution activities, and amino acid sequence) of isolated proteins.
Blinding	Blinding is not relevant to this study, because all the experimental data (FTIR, LC-MS, O2 evolution) are provided as direct outputs from the instruments in the individual measurements of isolated proteins.

Reporting for specific materials, systems and methods

We require information from authors about some types of materials, experimental systems and methods used in many studies. Here, indicate whether each material, system or method listed is relevant to your study. If you are not sure if a list item applies to your research, read the appropriate section before selecting a response.

Materials & experimental systems

n/a	Involved in the study	
☒	☐	Antibodies
☒	☐	Eukaryotic cell lines
☒	☐	Palaeontology and archaeology
☒	☐	Animals and other organisms
☒	☐	Human research participants
☒	☐	Clinical data
☒	☐	Dual use research of concern

Methods

n/a	Involved in the study	
☒	☐	ChIP-seq
☒	☐	Flow cytometry
☒	☐	MRI-based neuroimaging
